# A Passive and Wireless Sensor for Bone Plate Strain Monitoring

**DOI:** 10.3390/s17112635

**Published:** 2017-11-16

**Authors:** Yisong Tan, Jiale Hu, Limin Ren, Jianhua Zhu, Jiaqi Yang, Di Liu

**Affiliations:** School of Mechanical Engineering, Northeast Electric Power University, Jilin 132012, China; tanyisong@neepu.edu.cn (Y.T.); 2201500335@neepu.edu.cn (J.H.); 2201600247@neepu.edu.cn (J.Z.); 2201600365@neepu.edu.cn (J.Y.); 2201600281@neepu.edu.cn (D.L.)

**Keywords:** bone plate, tibia-bone plate-screw model, magnetic material, passive and wireless sensor, finite element analysis

## Abstract

This paper reports on a sensor for monitoring bone plate strain in real time. The detected bone plate strain could be used for judging the healing state of fractures in patients. The sensor consists of a magnetoelastic material, which can be wirelessly connected and passively embedded. In order to verify the effectiveness of the sensor, a tibia-bone plate-screw (TBS) model was established using the finite element analysis method. A variation of the bone plate strain was obtained via this model. A goat hindquarter tibia was selected as the bone fracture model in the experiment. The tibia was fixed on a high precision load platform and an external force was applied. Bone plate strain variation during the bone fracture healing process was acquired with sensing coils. Simulation results indicated that bone plate strain decreases as the bone gradually heals, which is consistent with the finite element analysis results. This validated the soundness of the sensor reported here. This sensor has wireless connections, no in vivo battery requirement, and long-term embedding. These results can be used not only for clinical practices of bone fracture healing, but also for bone fracture treatment and rehabilitation equipment design.

## 1. Introduction

Fracture is a common injury in the skeletal system, which happens at all ages and exerts a detrimental influence on human life. The key to successful bone unions is to use the appropriate fixation devices and accurate determination of the bone healing state. Many fixation devices, including external fixation, intramedullary nails, bone plates, wires, and pins, are used to restore diaphyseal fractures. Compared with other surgical interventions, internal fixations using bone plates show promising results. Therefore, bone plates are used widely in bone fracture treatments [1,2,3,4,5]. It is found in clinical work that most patients urge that fixation devices be taken out, so sound judgement about the healing state has a prominent influence on treatment results. At present, bone healing state detection depends upon periodical X-ray image examination after the bone plate is clinically implanted for treatment. This method relies on doctors’ clinical experience to a large extent. Once a wrong judgement is made and the bone plate is taken out prematurely, the bone fracture will not mend completely and the risk of future bone fractures will increase. On the contrary, if the bone plate is not taken out in time after the bone heals, the bone will grow excessively, which makes it difficult to remove the bone plate and locking screws. This leads to the retention of fixation devices [6,7]. In the early state of bone fracture healing using bone plates, the bone plate withstands most of the external force and endures greater stress. As the bone heals, the stress on the bone plate decreases gradually. Therefore, it is of great significance for bone fracture patients that judge bone healing state can be judged by detecting variations in bone stress.

## 2. Materials and Methods 

**Figure 1:**
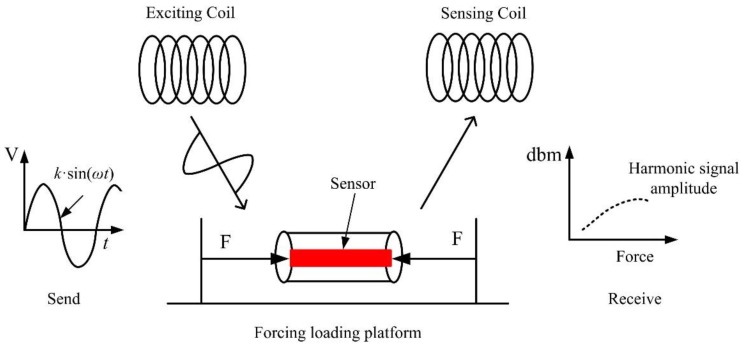
Schematic diagram of the sensor operation principle.

In this paper, we report on a passive and wireless sensor for in vivo bone plate strain monitoring. The implanted sensor operates in vivo without disrupting the patient’s normal activities and can be as short as a few hours or as long as a few years before it is removed. Meanwhile, the use of implantable sensors may also reduce the chance of developing infections since there is no need for potentially perturbing wires inside the body for data collection [8,9]. The sensing material is Fe_40_Ni_38_Mo_4_B_18_ (Model: 2826 MB, Metglas Inc., Hong Kong, China), which is a ribbon-like amorphous ferromagnetic alloy. The Fe_40_Ni_38_Mo_4_B_18_ alloy exhibits high permeability, low resistivity, a high magnetostriction coefficient, and high mechanical tensile strength [10,11,12]. The graphical function representation is shown in Figure 1. The sensor is excited by an exciting coil, which generates a magnetic field. When the sensor is subjected to a tension or compression strain, the permeability of the Fe_40_Ni_38_Mo_4_B_18_ alloy will change accordingly [13,14]. In response to the exciting field, the variation could be acquired by the sensing coil. During the whole process, no wires or batteries are needed for the sensor. Hence, it is ideal for long-term in vivo bone plate stress monitoring [15,16]. 

In order to verify the effectiveness of the sensor, a goat hindquarter tibia was selected for experiments. A bone plate was used to fix the bone fracture and a geometric model of the tibia-bone plate-screw (TBS) was established. The finite element method was utilized to analyze the stress variation when the bone heals. The ribbon-like sensor was stuck on the surface of the bone plate. The wounded bone together with the bone plate was mounted on the loading platform for force application. The stress on the bone could be detected via the sensing coil. The results of this paper can be used not only for clinical practices of bone fracture healing but also for bone fracture treatment and rehabilitation equipment design.

## 3. Experiments

### 3.1. The Tibia-Bone Plate–Screw Model

**Figure 2:**
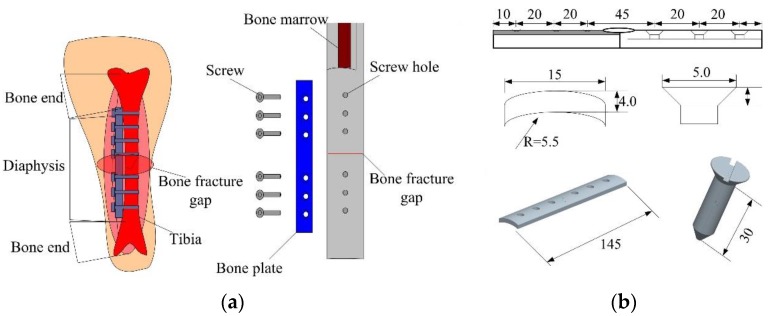
The tibia-bone plate-screw (TBS) model and dimensions. (**a**) the TBS model; (**b**) Dimensions of the bone plate.

The tibia is the bone, which supports the greatest weight of human beings. However, it is prone to damage from external crushes, crashes and strikes due to its weak subcutaneous tissues and muscles, as well as poor blood supply. Therefore, tibia fractures are a common orthopedic trauma, which has a high incidence rate [17,18,19]. The goat is a common quadruped mammal, which has firm and strong hindlimbs. The hindlimbs support the greatest weight of the goat, which is similar to that of human beings. Hence, the goat hindquarter tibia was selected for the experiment. The tibia of an adult healthy goat could be modeled as a cylinder. The Tibia-Bone Plate-Screw model is depicted in Figure 2. 

The tibia had a length of 300 mm and a diameter of 30 mm. The 2 mm wide transection thin layer represented the bone fracture. A commercially available non-locking compression bone plate (Model: 6-145-4, Huachuang Medical Apparatus & Instruments Inc., Guangzhou, China) with six holes was modeled and assembled with bone fragments with the help of six screws. The bone plate bridged the bone fragments and maintained enough space between the screws and fracture site to prevent interaction with injured tissues [20]. The detailed dimensions of bone plate and screws are given in Figure 2.

### 3.2. Finite Element Model Construction

**Figure 3:**
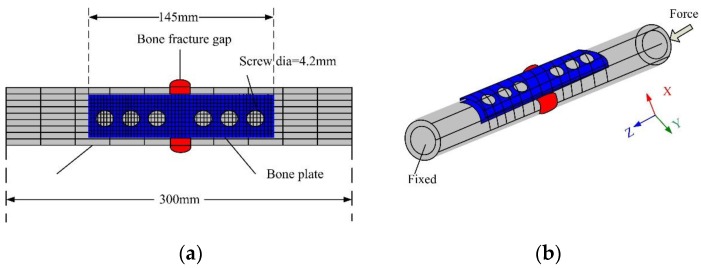
FEA mesh model and force loading condition of the TBS. (**a**) the FEA mesh model; (**b**) the TBS force loading condition.

Finite element analysis (FEA) is a mathematical approximation used for simulating the actual geometry and load conditions. FEA was first used in bone research by Brekelmans and Rybicki in 1972 [21,22]. It has strong adaptability, and the models are reusable. FEA was used in this paper in order to conduct accurate analysis of the force conditions of the tibia-bone plate-screw model. The FEA mesh was made first in the TBS model. The mesh element of the TBS was C3D20N, and an automated method was selected by the software. The numbers of units and numbers are shown in Table 1. Loading forces and constraint conditions were required for the established model in order to analyze force conditions during the healing of the goat hindquarter tibia, which is indicated in Figure 3b. The divided mesh model and force constraint conditions are given in Figure 3. The material properties used in FEA is shown in Table 1.

### 3.3. Experiment Preparation

The size of the bone plate used for sensor sticking is shown in Figure 2. As the mounting space of the bone plate is relatively small, the size requirement of the sensor is higher. The sensor used in this paper has a size of 10 mm × 10 mm × 30 µm (length × width × thickness). Seven pieces of the magnetoelastic material were stuck on the bone plate. Special attention should be paid to the selection of the adhesion used for sticking the sensor to the bone surface. Non-uniform stress would exist in the sensor if the glue or epoxy resin used for making the resistance strain gauge sensor was utilized in the bone sensor. Meanwhile, due to the different temperature coefficients between the sensor and the bone plate base, the temperature variation would result in non-uniform stress in the sensor. Accordingly, a high viscosity adhesive should be selected for the sensor to guarantee a rigid state between the bone plate and the sensor. Hence, a high stiffness adhesive J-B Weld was adopted when the sensor was being made. Furthermore, the sensor should be under non-stress conditions before the adhesive hardens completely. 

**Figure 4:**
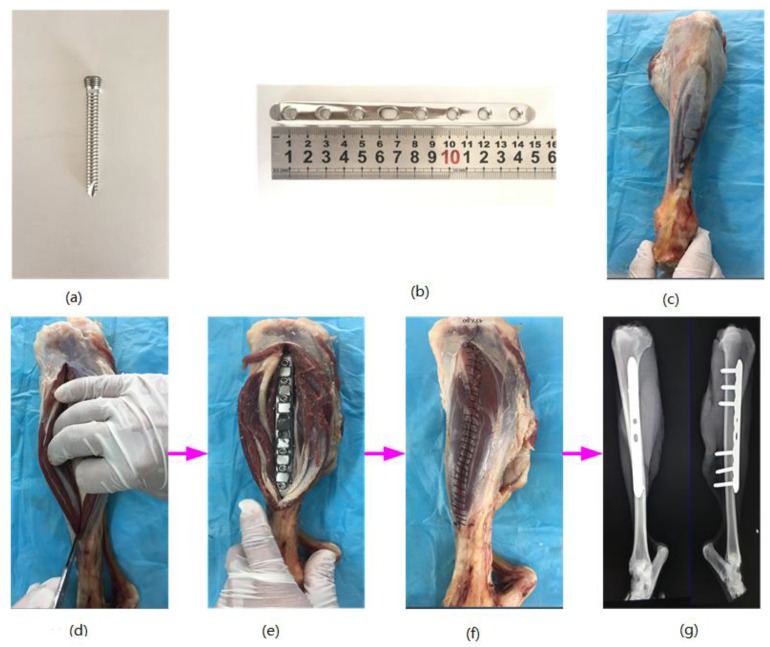
The installation process of the bone plate. (**a**) the bone plate screw; (**b**) the bone plate; (**c**) preparation; (**d**) cutting open; (**e**) implantation; (**f**) suture; (**g**) the X-ray image.

The surface tissues were cut open in the tibia region to allow the goat hindquarter tibia to be visible. The bone plate was attached to the outside of the tibia, and six locking screws were inserted into it successively to secure the bone plate and the tibia. After this, the wire saw was used to make an artificial bone fracture in the middle of the tibia. The wound was rinsed with iodophor and physiological saline before being sutured layer by layer. After this process, the bone plate fixation state was detected with X-rays. If no obvious displacement occurred, the bone plate was placed appropriately. Figure 4 shows the process of the bone plate installation and the X-ray film of the bone plate and tibia.

### 3.4. Experimental Setup

**Figure 5:**
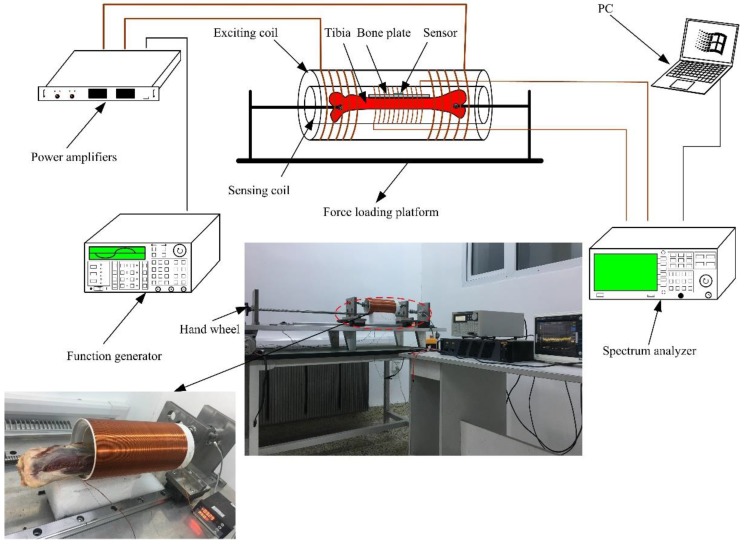
Sketch diagram of the experiment and prototype.

The graphical representation of the experiment is shown in Figure 5. The two coils are the exciting coil and sensing coil, respectively. The dimension of the exciting coil is 110 mm in diameter and 200 turns in length. The copper line used in the exciting coil is 0.5 mm in diameter. The dimension of the sensing coil is 90 mm in diameter and 200 turns in length. The copper line used in the sensing coil is 0.25 mm in diameter. The exciting coils generated alternating magnetic fields under the excitation of external sinusoidal sources. The sensor suffered from strain in the alternating magnetic field and generated an inverse magnetostriction effect, which resulted in the magnetic flux variation in the sensing coil. The sensing coil was linked to a spectrum analyzer to acquire the changing signals. The analyzed results were stored in a computer. 

In order to analyze the stress variation of the bone plate when the bone heals, a precise force should be loaded on it. Hence, a force loading platform was developed here, as shown in Figure 5. The bone was linked to two vertical plates via two universal joints at each of its end. The utilization of universal joints effectively eliminated any additional bending stress due to structure errors. The platform was equipped with high-precision load cells and could apply forces to the sensor via the hand wheel at its left. The loading platform was placed on a precision linear guide rail, which could reduce the frictional resistance while applying as much force as possible and ensure the high accuracy of the experiment. 

## 4. Results and Discussion

### 4.1. Analysis Results of the FEA Model

**Figure 6:**
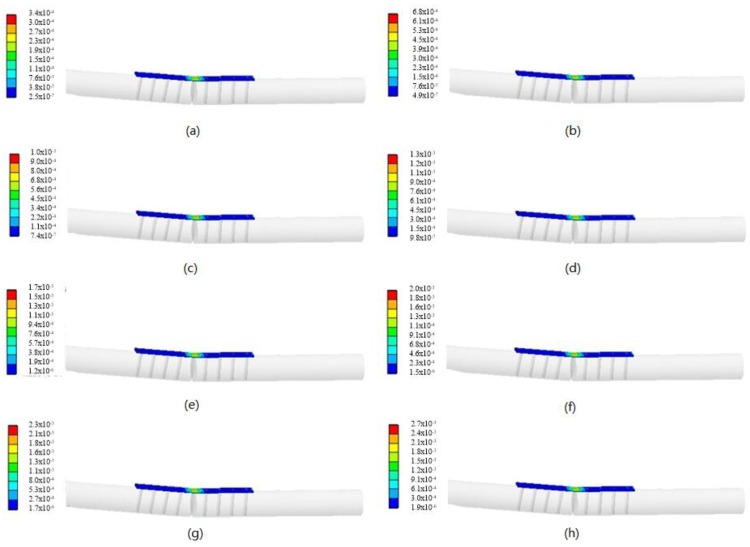
Strain distribution diagrams under different forces. (**a**) F = 50 N; (**b**) F = 100 N; (**c**) F = 150 N; (**d**) F = 200 N; (**e**) F = 250 N; (**f**) F = 300 N; (**g**) F = 350 N; (**h**) F = 400 N.

**Figure 7:**
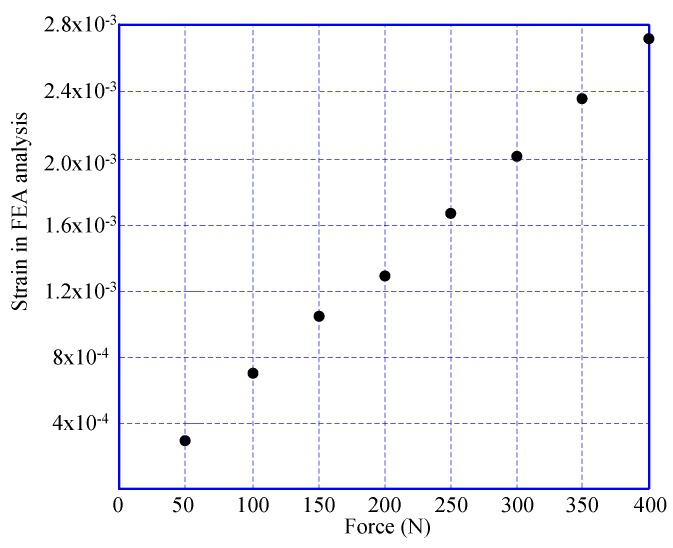
The relationship of external force and strain.

The supporting force provided by the bone plate decreased gradually as the tibia fracture healed itself from the FEA analysis. In order to simulate the change in the bone strain during the bone healing accurately, a force was exerted on the sensor, as shown in Figure 3. The force was evenly loaded at intervals of 50 N and the force range was 0–400 N (the goat in the experiment had a weight of about 40 kg). The material attributes of the TBS model are given in Table 1. Compressive strain distribution diagrams are illustrated in Figure 6. Figure 7 sketches the relationship between the external force and strain of the bone plate. It could be concluded that the strain in the bone plate increased gradually as the external force rose. In other words, the bone plate suffered from a larger strain at the early stage of the bone healing due to weak connections of the bone fracture region. After some time, the strain of the bone plate decreased step by step because the bone became stronger as it healed. This provides evidence that could be used aid in monitoring bone healing. 

### 4.2. Experimental Results of the TBS Prototype

**Figure 8:**
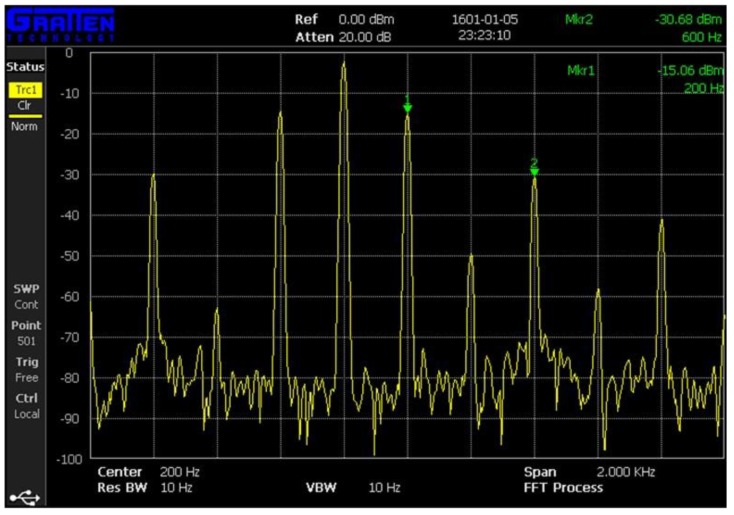
Results of the spectrum analyzer.

**Figure 9:**
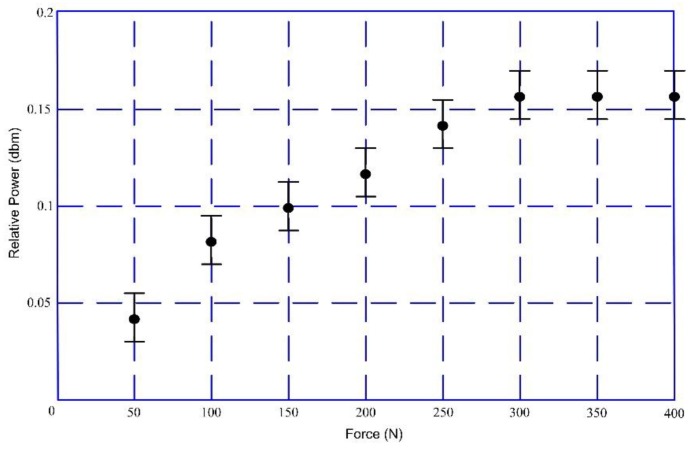
Relationship between external force and powers (exciting frequency: 200 Hz).

A sinusoidal signal with a frequency of 200 Hz and a voltage of 4 V was generated by a function generator (Fluke 271). The sinusoidal signal was used as the input into the exciting coil after a power amplifier (TAPCO Juice^TM^) was used to generate alternating magnetic fields. An external force was applied on the goat hindquarter tibia through the loading platform at even internals of 50 N. The force range was also 0–400 N. The bone plate withstood a compressive strain as the force exerted on bone became larger, which resulted in a strain on the magnetostriction material and a change in magnetic permeability accordingly. The magnetic permeability variation could be detected by the sensing coil. The analysis results by the spectrum analyzer (GA40XX) is depicted in Figure 8. From the results, we found that the 3rd harmonic frequency (600 Hz) had a larger amplitude and a more stable performance in the experiment. Hence, the 600 Hz harmonic frequency was selected as the detecting frequency for our experiment. Figure 9 shows the relationship between the external force and the powers of the detected 3rd harmonic signals. The power of the selected frequency increased as the external force became larger. Essentially, the strain on the bone plate increased at the same time. This allows for judgement of the bone healing state. After the force was 300 N, the output of the sensing coil did not change with the external force as the magnetostriction materials gradually became saturated (the saturation magnetostriction of the sensor is 12 ppm).

### 4.3. Discussion

**Figure 10:**
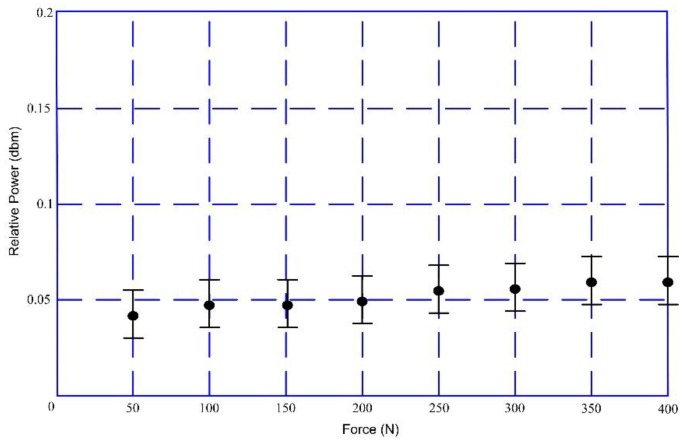
The relationship between external force and powers (exciting frequency: 400 Hz).

The key concern of this paper is to judge the healing state accurately according to the bone plate strain in different healing stages to improve the healing rate of the bone fracture when fracture patients are treated with internal bone plate fixation. An accurate TBS model has an important influence on the experiments. The key part of this experiment is the sensor selection. The magnetostriction material stability, a wireless connection, and passive embedding. It can work on the bone plate in the long term, which allows it to work in the bone fracture region as well. Due to the high sensitivity of the sensor, the final data may have relatively large differences due to diverse exciting signal frequencies and amplitudes. During the experiments, we used exciting signals with frequencies of 200 Hz, 400 Hz, 600 Hz, and 800 Hz, respectively. The 200 Hz signal was found to have the best result. Figure 10 is the output curve under the 400 Hz exciting signal. From the comparison between Figure 9 and Figure 10, the output under the 400 Hz exciting signal was found to have little variation and the output was very hard to distinguish. Hence, the experimental results depended on exciting signal frequencies. Essentially, there is no uniform standard in bone plate strain and output power. Finally, there is no explicit theoretical guidance on how to select the optimum excitation signal, which we are studying now.

## 5. Conclusions

An intelligent bone plate based on the magnetostriction material Metglas 2826 MB was designed, calculated, fabricated, and tested in this study. The relationship between the stain and applied force was determined by FEA calculation. The relationship between the sensor output (dbm) and the applied force was established in the actual platform. After this, the relationship between the stain and the sensor output was indirectly acquired through the applied force. The sensor could monitor the healing state of the damaged bone when the bone recovered. Compared with a standard strain gauge, which needs wires for connection, the intelligent bone plate can achieve obvious improvements in wireless and passive applications. Future work will focus on finding the optimal exciting signal frequency and practical bone fracture modelling.

## Figures and Tables

**Table 1:** Details of finite element analysis (FEA) model and material properties used in FEA.

FEA Model	Elements/Odes	Material Properties		
		Young’s Modulus (GPa) Tensile Strength (GPa) Poisson’s Ratio		
Tibia	7475/13,216	14	0.117	0.488
Bone plate	5309/9750	193	0.52	0.31
Screw	2101/3630	193	0.52	0.31
